# Fatigue assessment by FACIT-F scale in Pakistani cohort with Rheumatoid Arthritis (FAF-RA) study

**DOI:** 10.12669/pjms.37.4.3602

**Published:** 2021

**Authors:** Abrar Ahmed Wagan, Abdul Raheem, Afra Bhatti, Taimoor Zafar

**Affiliations:** 1 Dr. Abrar Ahmed Wagan, MBBS, FCPS (Medicine), FCPS (Rheumatology), FACR. Assistant Professor, Indus Medical College, Tando Mohammad Khan, Pakistan; 2 Dr. Abdul Raheem, MBBS. Postgraduate Trainee, Central Park Medical College Lahore, Lahore, Pakistan; 3 Dr. Afra Bhatti, MBBS. Postgraduate Trainee, Central Park Medical College Lahore, Lahore, Pakistan; 4 Dr. Taimoor Zafar, MBBS. Postgraduate Trainee, Central Park Medical College Lahore, Lahore, Pakistan

**Keywords:** Fatigue, Das-28, RA, FACIT-F score, HCV

## Abstract

**Objective:**

To fine out fatigue frequency and severity by FACIT-F scale in Pakistani cohort with rheumatoid arthritis.

**Methods:**

This study was conducted at department of Medicine division of rheumatology CPMC Lahore. After the approval of IRB, 192 patients of RA were recruited. Written, informed consent was taken, demographic details were noted, patients filled the URDU version of FACIT-F (fatigue severity scale). 5-ml of blood was taken for fasting blood sugar, viral markers and ESR by a trained phlebotomist. Each individual’s disease activity was assessed by DAS-28 and FACIT-F score was calculated.

**Results:**

The Mean age (39.9±10.5) years, (71.9%) were females. Fatigue frequency was 62% (n=126), age, education, hypertension, DAS-28, exercise levels and HCV gives significant association with fatigue score. Linear regression analysis, results showed one unit increase in DAS-28 will gives 2.71 unit increases in fatigue scores(P <0.05).

**Conclusions:**

We have very high frequency of fatigue in RA, increases with disease activity & associated conditions.

## INTRODUCTION

Rheumatoid arthritis is an autoimmune disease prevalent mostly in young to middle-aged women, with hallmark features of synovitis leading to articular cartilage - bone destruction, deformities, osteoporosis, and constitutional features.^1^ Fatigue is perceived as lack of energy, leading to disruption of daily activities and it is widely present in all autoimmune disorders. Fatigue in RA is due to multiple reasons like: Pain, mental stress, depression, disease activity, medications and disability, its severity varies with different times of day.^2^

Unlike normal day to day tiredness, fatigue in RA is chronic, not related to overexertion and it persists even after adequate rest. It’s an important physical and cognitive symptom which is difficult to control, very unpredictable, overwhelming, and affects every aspect of life.^3^ In recent Brazilian study the frequency of fatigue was estimated around 71.25% while overall range between 40% to 80%.^4^,^5^ Dupond et al, also reported near similar frequency range of 20% to70% and described psychological fatigue or weariness as most common pattern, and depression being the most common cause of fatigue in inflammatory rheumatic diseases.^6^

Minnock et al, did a longitudinal study with RA patients (n = 87) to know the correlation of fatigue and disease activity, results revealed fatigue is not explained by disease activity as represented by the ACR core set outcomes, rather it’s a behavioral variable with multifactorial influences.^7^

Trine Pilgaard et al, in most recent study of 633 patients with three major autoimmune diseases, RA,ankylosing spondyloarthritis and psoriatic arthritis found fatigue frequency of 61% and interestingly they used most recent FACIT-F questionnaire for fatigue assessment.^8^

Assessment of fatigue is problematic due to lack of an objective marker, albeit there are more than 12 patient-reported outcome measures (PROMs) such as Profile of Mood States, Short Form 36 (SF-36), Multidimensional Assessment of Fatigue, Ordinal Scales, Visual Analog Scales, and Functional Assessment of Chronic Illness Therapy-Fatigue (FACIT-F).^9^ FACIT-F, invented in 1997, covers physical, functional and emotional fatigue, also assess its social consequences with good internal consistency and reliability.^10^

Worldwide more focus has been given to fatigue and its socio-economic impact; through this study we have tried to assess the fatigue frequency in local population and its association with other conditions.

## METHODS

After the approval of IRB(CPMC/IRB/1723) this cross sectional study was conducted at Central Park Medical College outpatient department of medicine division of rheumatology. Written and informed consent was taken from each participant. A sample size of 192 cases was calculated.

Seropositive (RA factor and anti-CCP antibody) RA patients were included. Sero-negative RA,SLE, Scleroderma, Sjogren syndrome, MCTD, psoriatic arthritis, osteoarthritis, primary or secondary fibromyalgia, polymyalgia rheumatic, physical disability, hypothyroidism, history of chemotherapy or radiotherapy in last two years, use of biological DMARDs, known cases major depression, panic disorders, somatization disorders, injudicious use of following medications (hypnotics, muscle relaxants, antidepressants, first-generation antihistamines, beta-blockers, opioids), chronic diseases were excluded.

A questionnaire was used to collect demographic data; it included outcomes such as years since diagnosis, marital status, education, employment status, exercise level, social status and smoking habits. Each participant BMI and Blood pressure was measured as per laid down protocols. Afterward 5 ml of blood was drawn by a trained phlebotomist for CBC, ESR, fasting blood sugar levels, Hbs Ag and anti HCV Antibody test. Fatigue assessment we used FACIT-F scale (URDU-version), its permission was sought prior to its use. The FACIT-Fatigue scale is a 13-item questionnaire assessing self-reported fatigue and the total score ranges from 0 to 52. For analysis of severity of fatigue, scores were categorized into four grades: composite score 40-52=little or no fatigue, score: 27-39=some fatigue, score: 14-26 =quite a lot of fatigue, score: 0-13=extreme fatigue. Rheumatoid arthritis disease activity score was calculated by DAS-28 calculator. Each study patient was examined by a senior physician.

### Statistical Analysis

Analysis was done by using IBM-SPSS version 23.0. Count with percentages given for qualitative characteristics, Mean with standard deviation given for quantitative characteristics of samples. Pearson Chi Square test was used to check the association of fatigue scores with studied parameters. Pearson Correlation analysis was done to see the relationship of fatigue score with body mass index, SBP, DBP, FBS and DAS. Linear regression analysis was done to design a model to analyze the dependency of fatigue score on SBP, DBP and disease activity score after adjusting for age and BMI, p-values less than 0.05 were considered significant.

## RESULTS

There were 192 samples with mean age (39.9±10.5)years, (47.9%) were aged between 31 – 45 years old, (71.9%) female gender, (45.3%) obese , the mean BMI was 30.1±6.3 kg/m2 , (80.7%)married samples, (25.5%)secondary and higher educated, (56.8%)belongs to poor working class, (35.9%)middle class, (76%) nonsmoker, (21.4%) were doing regular exercise,(60.4%) never performed exercise, (33.9%) samples had DAS-28 under 2.6 defined as disease remission, (10.4%) were HCV positive and (2.6%) Hbs-Ag positive. Table-I.

**Table-I:** Baseline Characteristics of Study Samples (n= 192).

Variables		n	%
Age Group (years)	<=30 years	44	22.9
	31 - 45 years	92	47.9
	>45 years	56	29.2
	***Mean ± SD***	*39.9±10.5*	
Sex	Female	138	71.9
	Male	54	28.1
BMI Levels (kg/m^2^)	<24.9:Normal weight	39	20.3
	25 - 29.9:over weight	66	34.4
	>30 Obese	87	45.3
	***Mean ± SD***	*30.1±6.3*	
Marital Status	Married	155	80.7
	Unmarried	26	13.5
	Other	11	5.7
Education	Uneducated	78	40.6
	Primary	17	8.9
	Secondary and higher	49	25.5
	Bachelors and beyond	48	25.0
Social Status	Upper	14	7.3
	Middle	69	35.9
	Working	32	16.7
	Poor	77	40.1
Smoking	Smoker	46	24.0
	Nonsmoker	146	76.0
Hypertension	Yes	37	19.3
Exercise Level	Regular	41	21.4
	Never	116	60.4
	Occasional	35	18.2
DAS-28	<2.6 : Remission	65	33.9
	2.6-3.2 : Low	44	22.9
	3.2 - 5.1 : Moderate	61	31.8
	>5.1: High	22	11.5
	***Mean ± SD***	*3.4±1.2*	
HCV	Positive	20	10.4
Hbs	Positive	5	2.6

The mean disease duration was (6.85±4.39) years, mean weight was (80.42±15.52) kg, mean height (64.59±3.55) inches, mean SBP (121.45±17.40) mmHg, mean DBP (79.48±9.0) mmHg, mean FBS (104.25±29.73) mg/dl, mean DAS-28 score (3.4±1.2), and mean fatigue scores was (34.94±8.88) units. Table-II.

**Table-II:** Baseline Quantitative Parameters.

Parameters	Mean	SD
Duration of disease	6.85	4.29
Weight (kg)	80.42	15.52
Height (inches)	64.59	3.55
SBP	121.45	17.40
DBP	79.48	9.0
FBS	104.25	29.73
Fatigue score	34.94	8.88

Fatigue frequency was 62% (n=126), some fatigue was present in 45.8% (n=88), quiet allot of fatigue in 19.3% (n=37) and extreme fatigue 0.5% (n=1) Bar Chart-1.

While association of fatigue scores with studied parameters, results showed, age , education, hypertension, DAS-28, exercise levels and HCV gives significant association with fatigue scores levels, whereas there was no significant association obtained for gender, Marital Status, BMI levels, smoking, social status and Hbs. Table-III.

**Table-III:** Association of Fatigue with Studied Parameters Using Pearson Chi Square test.

Characteristics		No or little fatigue (n=66)		Some fatigue (n=88)		Quite a lot fatigue (n=37)		p-value

		n	%	n	%	n	%	
Age Group (years)	<=30 years	22	33.3	12	13.6	10	27.0	<0.01*
	31 - 45 years	34	51.5	50	56.8	8	21.6	
	>45 years	10	15.2	26	29.5	19	51.4	
Sex	Male	50	75.8	61	69.3	26	70.3	0.74
Marital Status	Married	52	78.8	71	80.7	31	83.8	0.10
Education	Uneducated	15	22.7	35	39.8	28	75.7	<0.01*
	Primary	6	9.1	10	11.4	0	0.0	
	Secondary and higher	27	40.9	19	21.6	3	8.1	
	Bachelors and beyond	18	27.3	24	27.3	6	16.2	
BMI Levels	<24.9:Normal weight	18	27.3	15	17.0	6	16.2	0.54
	25 - 29.9:over weight	23	34.8	29	33.0	14	37.8	
	>30 Obese	25	37.9	44	50.0	17	45.9	
smoking	smoker	53	80.3	65	73.9	27	73.0	0.70
Social Status	Upper	5	7.6	6	6.8	3	8.1	0.11
	Middle	29	43.9	31	35.2	9	24.3	
	Working	13	19.7	17	19.3	2	5.4	
	Poor	19	28.8	34	38.6	23	62.2	
Hypertension	Yes	7	10.6	16	18.2	14	37.8	<0.01*
DAS-28	<2.6 : Remission	12	18.2	33	37.5	19	51.4	<0.01*
	2.6-3.2 : Low	5	7.6	27	30.7	12	32.4	
	3.2 - 5.1 : Moderate	33	50.0	23	26.1	5	13.5	
	>5.1: High	16	24.2	5	5.7	1	2.7	
Exercise level.	Regular	19	28.8	17	19.3	5	13.5	0.04*
	Never	31	47.0	54	61.4	30	81.1	
	Occasional	16	24.2	17	19.3	2	5.4	
HCV	Positive	3	4.5	7	8.0	10	27.0	<0.01*
Hbs	Positive	1	1.5	1	1.1	3	8.1	0.13

* p<0.05 was considered significant using Pearson Chi Square test.

Correlation analysis of fatigue scores with quantitative parameters, results showed body mass index gives 15.1% negative relationship with fatigue scores, DBP was also 14.8% negatively associated, and DAS-28 gives 42.6% positive association with fatigue scores. These correlations found statistically significant with (< 0.05). Table-IV.

**Table-IV:** Correlation Analysis of Fatigue Scores.

Parameters	R-value	R-square (%)	p-value
Duration of disease	-0.052	0.27	0.475
BMI (kg/m2)	-0.151	2.28	0.036*
SBP	-0.197	3.88	0.006*
DBP	-0.148	2.19	0.04*
FBS	-0.122	1.49	0.091
Disease Activity score	0.426	18.15	<0.01*

* p<0.05 was considered significant.

Linear regression analysis, results showed one unit increase in DAS-28 will gives 2.71 unit increases in fatigue scores on average when SBP, DBP kept constant and model adjusted for age and BMI. Table-V.

**Table-V:** Effect of Studied Parameters on Fatigue Score.

Independent Parameters	Beta Coefficients		t-value	p-value	95.0% Confidence Interval for B	
	
	B	S.E			Lower Bound	Upper Bound
SBP	-0.03	0.05	-.619	0.53	-0.13	0.07
DBP	0.03	0.09	.395	0.69	-0.15	0.22
Disease Activity score	2.71	0.46	5.894	<0.01*	1.80	3.61

Dependent Variable: Fatigue score: Model were adjusted for Age and BMI ,

* P<0.05 was considered significant.

**Figure:**
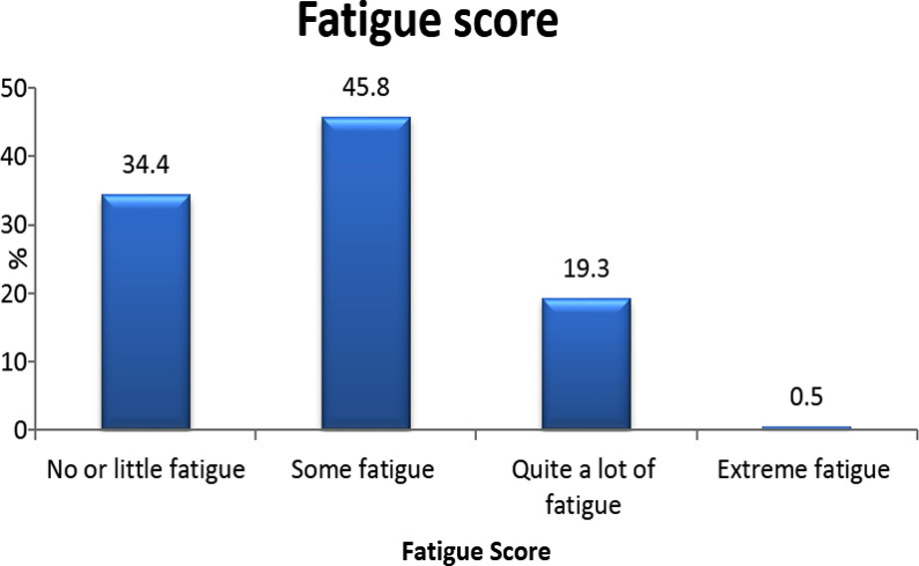
Bar Chart :1

## DISCUSSION

Fatigue in Rheumatoid arthritis is a common complaint which exerts immense burden over the person’s life, impairs the quality of living and incur huge financial losses. Abdel Moneim H. Helal et al^11^ in case control study found that fatigue is more prevalent in RA patients who had raised serum levels of interleukin-6, independent of disease duration and activity. In our study results we found there was direct relationship between disease activity and fatigue score, Dhir et al also reported same it is significantly associated with high DAS28 scores.^12^

Cécile L. Overman et al in his international sample study described the frequency of fatigue in various rheumatic diseases and reported 41 to 57% of patients with rheumatoid arthritis had severe fatigue and in our study, we found frequency of 63.2%.^13^

Fatigue taken as important extra-articular manifestation of RA, related to disease activity, there are multiple causes for it, including disability, sound mental health, social support and wellbeing, it has been cause of various deleterious effects in a patient’s life like: leads to role limitations in (36-44%), physical and social issues in (52-57%), reason of mental health effects in (64%), and most importantly being the prime reason for perception of worsening of health (51%).^14^

Fatigue incurs some harmful effects on persons daily living like: activity impairment (OR 1.52; 95% CI: 0.79, 2.26) and work productivity loss (OR 4.16; 95% CI: 2.47, 5.85).^15^ In patients where RA is difficult to control labeled as (difficult to treat) fatigue might the biggest contributor, making treatment plans more challenging and ultimately more health care load.^16^ Fatigue causes problems in assessment of disease activity, considered as the main factor which affects the components disease activity scales like self-assessment of global impact of the disease (patient global assessment), due to it persistence patients might not get the benefits of better disease control despite of disease remission.^17^,^18^

We have assessed the severity of fatigue in established RA patients, its socioeconomic determinants and looked into the impact of anti hcv and Hbs Ag status over fatigue, prior to this no study has seen towards this side as these chronic hepatitis is very common in south Asian countries.In current study the mean fatigue scores was (34.94±8.88) units, Trine P et al^8^ also found near similar results with mean score of (34.86 ±11.04) but our study population was much younger.

Its pathophysiology is linked to disease activity, persistent pain, sleep deprivation, mood disturbances, other diseases, high inflammatory markers, elevated cytokines, with these a person feel tired or even exhausted similarly to prodrome of an infection.^19^ Fatigue has been linked to conventional DMARDs use, specially methotrexate and sulfasalazine, this may contribute to non-adherence to their use, so change in mode of administration and dosing regimen may help.^20^,^21^Fatigue has severe financial impact leading to very high clinical care costs, increasing consultations, key determinant of sickness, absence and loss of employment, despite of its perceived importance and increased research activity, our understanding for prognostic factors of poor fatigue outcomes is lacking, ultimately poorly managed.^22^

Curbing fatigue is possible, by multidimensional approach (non-pharmacologic and pharmacologic) measures, like exercise, cognitive behavioral therapy ,timely and effective use of conventional and biological dmards especially IL-6 blocker (Tocizilumab) and newer medications called conventional synthetic dmards Janus kinase (JAK) inhibitors (tofacinitab, baricinitab),in active, early RA, methotrexate naive, methotrexate and biologics inadequate responders shown major improvements, also treatment of anemia, Vit D and Omega 3 fatty acids supplementation may help.^19^

## CONCLUSION

Fatigue in RA is complex entity, over all it has a negative impact on person’s wellbeing. Fatigue needs to be addressed and assessed at least twice a year with validated tool. Its management in terms of early diagnosis and treatment is very important as this may lead noncompliance of some very basic and essential medications required for control. This may be always considered in those who couldn’t reach treat to target goals.

### Limitations of the Study

This is a cross sectional study, with small sample size, results can’t be generalized, more longitudinal studies are required to know the dynamics of fatigue, frequency and management, while this is the first study of its kind done on local population, used a fatigue assessment tool which is translated in local language and easy to understand.

### Authors Contribution:

**AAW:** Design, drafting, Data acquisition data analysis, data interpretation, final approval.

**AR, AB, and TZ:** Data acquisition, data analysis, interpretation, drafting, final approval.
